# Clinical outcomes and management of idiopathic retroperitoneal fibrosis: a case series

**DOI:** 10.1097/MS9.0000000000004780

**Published:** 2026-04-29

**Authors:** Ahmed Mansour Alkhunaizi, Mahmoud Ahmed, Ali Alshaqaq

**Affiliations:** Division of Nephrology, Johns Hopkins Aramco Healthcare, Dhahran, Saudi Arabia

**Keywords:** autoimmune fibrosis, case series, fibrosis regression, hydronephrosis, idiopathic retroperitoneal fibrosis, ureteral obstruction, urology

## Abstract

**Background:**

Idiopathic retroperitoneal fibrosis (IRF) is a rare fibro-inflammatory disorder that often leads to ureteral obstruction and renal impairment. This case series evaluated clinical presentation, diagnostic evaluation, management strategies, and outcomes in nine patients diagnosed with IRF at a single tertiary center.

**Methods:**

We retrospectively reviewed nine patients diagnosed with IRF between January 2015 and December 2024. Clinical presentation, comorbidities, laboratory parameters, imaging findings, targeted immunologic and pathology tests, treatment regimens, and longitudinal outcomes were extracted from electronic medical records

**Results:**

All nine patients (100%) presented with hydronephrosis, bilateral in eight (89%) and unilateral in one (11%). Biopsy to exclude malignancy was performed in six patients (67%). Medical therapy comprised prednisone alone or in combination with mycophenolate mofetil in six (67%) and azathioprine in two (22%). Ureteral stenting was performed in seven patients (78%). Clinical improvement occurred in eight patients (89%), radiologic improvement in six (67%), and renal function stabilized or improved in all cases (100%). Complications occurred in five patients (56%): acute kidney injury in two (22%), persistent unilateral renal nonfunction in two (22%), and chronic kidney disease in one (11%).

**Conclusion:**

IRF posed significant diagnostic and therapeutic challenges. Early use of multimodal imaging enabled prompt diagnosis, and combined corticosteroid plus immunosuppressive therapy achieved high rates of clinical response, but was associated with notable morbidity, underscoring individualized management and close monitoring.

## Introduction

Idiopathic retroperitoneal fibrosis (IRF) is a rare fibro-inflammatory disorder characterized by dense fibrotic tissue in the retroperitoneum, often encasing adjacent structures such as the ureters and great vessels. First described by Ormond in 1948, IRF accounts for approximately two-thirds of all retroperitoneal fibrosis cases, with the remainder attributed to secondary causes such as malignancy, infections, IgG4-related disease, or medications^[^1–4^]^.

The pathogenesis of IRF is believed to be immune-mediated, with associations to autoimmune diseases and elevated levels of pro-inflammatory cytokines such as interleukin-6 and interleukin-13^[^1^]^. Genetic predispositions, including HLA-DRB1*03, and environmental exposures like asbestos and tobacco smoke have also been implicated^[^5,6^]^. Clinically, IRF presents insidiously with nonspecific symptoms such as abdominal or flank pain, weight loss, and fatigue. Ureteral obstruction is a hallmark complication, potentially leading to hydronephrosis and renal dysfunction.

Diagnosis typically relies on imaging modalities such as excretory urography, sonography, contrast-enhanced computed tomography (CT), or magnetic resonance imaging (MRI), which reveal a retroperitoneal mass encasing the aorta and/or ureters^[^7^]^. Histopathological confirmation may be necessary to exclude malignancy. Treatment strategies include immunosuppressive therapy most commonly glucocorticoids, often combined with agents like methotrexate or mycophenolate mofetil (MMF)^[^8^]^. In refractory cases, biologics such as rituximab have shown promise^[^9,10^]^. Surgical interventions, including ureterolysis or stenting, are reserved for significant obstruction or poor response to medical therapy.

This study presented observational data on nine patients with IRF treated at our institution, detailing their clinical presentations, management strategies, and outcomes to illuminate the significant diagnostic and therapeutic challenges of this rare disease.

## Methods

### Study design and setting

We conducted a retrospective case series of nine patients diagnosed with IRF at Johns Hopkins Aramco Healthcare, a tertiary care center, between January 2015 and December 2024. Institutional ethics committee approval was obtained (Approval #25-06-223). All data were de-identified prior to analysis: direct identifiers (names, medical record numbers, full dates of birth, and addresses) were removed, each subject was assigned a unique study ID, and dates were reported as age or month/year to minimize re-identification risk. The ethics committee waived the requirement for informed consent for use of retrospective, anonymized data.

#### Patient selection and diagnostic approach

Patients were included when clinical presentation and imaging were consistent with retroperitoneal fibrosis and secondary causes were excluded by targeted evaluation. Secondary causes considered and actively excluded included malignancy, infection, prior radiation, drug-induced fibrosis, and IgG4-related disease when clinically suspected. Imaging modalities included renal and abdominal ultrasonography, contrast-enhanced CT or MRI and positron emission tomography (PET) scan. Biopsy for histopathology was not performed when imaging features were classic and the clinical course was concordant or when biopsy was contraindicated or refused.

#### Data collection and definitions

We extracted demographics, comorbidities, smoking status, occupational or environmental exposures, prior autoimmune diagnoses, baseline medications, presenting symptoms and symptom duration, baseline and follow-up renal function (serum creatinine, eGFR), inflammatory markers (ESR, CRP), autoimmune serologies (ANA, IgG4 when available), imaging findings including baseline mass dimensions and hydronephrosis grade, histopathology results, treatments (medical and procedural), treatment duration, complications, and recurrence. Missing data reflected unavailable external referral records or tests not performed and were recorded as “Not documented.” Clinical improvement was defined as resolution or marked reduction of presenting symptoms. Radiologic improvement was defined as documented reduction in mass size or extent or improvement/resolution of hydronephrosis on follow-up imaging.

#### Treatment protocols and follow-up

Prednisone was initiated at 1 mg/kg/day and tapered over 6 months in most patients. MMF was given at 1000 mg twice daily; azathioprine was dosed at 1.5 mg/kg/day. Treatment duration was individualized between 12–24 months based on clinical response, radiologic regression, inflammatory marker trajectory, and tolerability. Patients with relapse or incomplete radiologic regression after 12 months were continued to 18–24 months. Ureteral stenting was performed for obstructive uropathy as indicated by imaging or renal compromise. Follow-up imaging and laboratory surveillance were performed per treating team discretion.

Follow-up duration was calculated as the interval from the year of diagnosis (derived from year of birth plus age at diagnosis) to the end of the study period (31 December 2024); when the computed diagnosis year preceded January 2015, diagnosis year was set to 2015 to reflect inclusion criteria and ensure consistent cohort ascertainment.

This case series has been reported in line with the PROCESS Guideline^[^11,12^]^.

### Statistical analysis

Descriptive statistics were used. Continuous variables were reported as medians with interquartile ranges and categorical variables as frequencies and percentages.

HIGHLIGHTS

- All patients with idiopathic retroperitoneal fibrosis presented with hydronephrosis (100%), predominantly bilateral (89%), confirmed by sonography and/or CT/MRI.
- Immunosuppressive therapy: prednisone alone or with mycophenolate mofetil (MMF) in 67%; azathioprine in 22%; ureteral stenting performed in 78%.
- Clinical improvement achieved in 89%; radiologic regression observed in 67%; renal function stabilized or improved in 100%.
- A self-limiting course is possible.
- Treatment-related complications occurred in 56%, including acute kidney injury (AKI) (22%), persistent unilateral renal nonfunction (22%), and chronic kidney disease (CKD) (11%).
- Emphasis on the importance of early multimodal imaging for prompt diagnosis, combined corticosteroid–immunosuppressive regimens for high response rates, and vigilant long-term follow-up to mitigate complications.
- Prednisone + MMF yielded an 89% clinical response in idiopathic retroperitoneal fibrosis.
- Multimodal imaging enabled early diagnosis and guided timely ureteral stenting.
- Despite high response, 56% experienced complications: AKI, CKD, or renal nonfunction.
- A self-limiting course of IRF is possible.

## Results

### Cohort characteristics and presentation

Nine patients were included with a median age at time of diagnosis of 52 years (IQR 18.5–61.5); two patients were 14 years old. (Table 1). Median follow-up was 84 months (IQR 48–108). Comorbidities included hypertension (n = 3), diabetes mellitus (n = 2), and ischemic heart disease (n = 1). Smoking history was present in two patients; no recognized occupational or environmental exposures associated with secondary retroperitoneal fibrosis were identified. One patient had history of Guillain-Barre syndrome. One pediatric patient had intrauterine growth retardation, growth hormone deficiency, bronchopulmonary dysplasia, angioedema, juvenile idiopathic arthritis and underwent total hip arthroplasty at a young age. She received treatment with Somatropin and Etanercept. No other established autoimmune disorders were documented. The second pediatric patient was otherwise a healthy child with no significant comorbidities. Baseline nephrotoxic medications were absent in the cohort. Flank or abdominal pain was the predominant presenting symptom in eight patients (89%). Constitutional symptoms (fatigue, weight loss) occurred in four patients (44%). Symptom duration ranged from 2 weeks to 12 months; duration was not documented in two cases.

**Table 1:** Summary of clinical features, diagnostic findings, treatments, and outcomes in nine patients with idiopathic retroperitoneal fibrosis.

Case	Age at diagnosis (Years)	Sex	Duration of symptoms	Serum Cr (Baseline→FU)	ESR/CRP	Imaging	Biopsy	Treatment	Clinical Outcome	Complications
C1	14	F	ND	0.6 → 0.7	NA	Bilateral hydronephrosis	No	Ur.stenting + steroids + MMF	Improved	None
C2	59	M	12 mo	0.8 → 0.8	Normal	Bilateral hydronephrosis	No	Steroids + MMF	Improved	None
C3	52	F	7 mo	0.9 → 1.0	Normal	Bilateral hydronephrosis	Yes	Steroids + Aza/MMF	Improved	None
C4	50	M	2 mo	1.7 → 1.0	High	Bilateral hydronephrosis	Yes	Ur.stenting + steroids + MMF	Improved	AKI
C5	70	M	7 mo	1.8 → 1.4	High	Right hydronephrosis	Yes	Ur.stenting + steroids	Improved	AKI
C6	14	F	5 mo	4.5 → 0.8	High	Bilateral hydronephrosis	Yes	Ur.stenting + steroids + MMF	Improved	Nonfunctioning Lt. kidney
C7	59	M	2 wks	5.0 → 0.9	High	Bilateral hydronephrosis	No	Ur.stenting + steroids + MMF	Improved	Nonfunctioning Lt. kidney
C8	23	F	4 mo	0.4 → 0.6	NA	Bilateral hydronephrosis	Yes	Ur.stenting	Improved	None
C9	64	M	ND	3.8 → 2.5	High	Bilateral hydronephrosis	Yes	Ur.stenting + steroids + Aza	Variable	CKD

AKI, acute kidney injury; Aza, azathioprine; CKD, chronic kidney disease; Cr, creatinine mg/dl; CRP, C-reactive protein; ESR, erythrocyte sedimentation rate; FU, follow-up; Lt, left; MMF, mycophenolate mofetil; mo, months; ND, not documented; Ur.stenting, ureteral stenting; wks, weeks.

### Diagnostic evaluation

**Figure 1.**
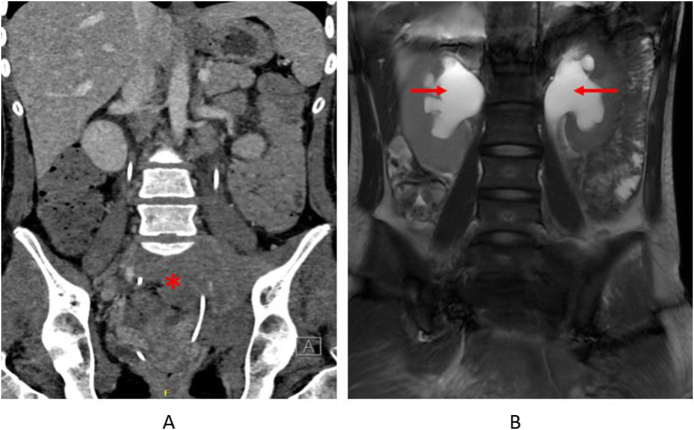
Imaging findings in one of the pediatric patients. (A) Abdominal CT scan showing a pelvic mass (asterisk). (B) MRI demonstrating bilateral hydronephrosis (arrow).

All patients underwent sonography and contrast-enhanced CT and/or MRI confirming retroperitoneal soft tissue encasing ureters or vascular structures. One patient underwent PET scan and that showed high fluorodeoxyglucose uptake in one abdominal area and no signs of lymph node involvement. Figure 1 shows the magnitude of the fibrotic mass and the severity of hydronephrosis in one of the pediatric cases. Biopsy was performed in six patients (67%) to exclude malignancy and to characterize histopathology. One of the pediatric patients underwent a second biopsy to rule out malignancy at another institution. The biopsy demonstrated atypical spindle cell proliferation of uncertain line of differentiation and associated fibrosis. In three patients, biopsy was not performed due to either classic imaging appearances with typical distribution and stable clinical course (*n* = 2) or patient refusal/contraindication to invasive sampling (*n* = 1). Inflammatory markers (ESR or CRP) were available in seven patients and were elevated in five (71%); inflammatory marker results were not documented in two patients.

### Treatment and clinical course

Prednisone was initiated at 1 mg/kg/day and tapered over 6 months in most patients. Mycophenolate mofetil at 1000 mg twice daily was combined with prednisone in six patients (67%); azathioprine at 1.5 mg/kg/day was used in two patients (22%). One patient’s medication was switched from MMF to azathioprine due to adverse effects. One patient declined immunosuppressive therapy and was monitored after ureteral stenting. She remained stable with regression of hydronephrosis upon follow-up. Treatment duration was individualized between 12 and 24 months based on clinical response, radiologic regression, inflammatory marker trajectory, and tolerability. Patients with relapse or incomplete radiologic regression after 12 months were continued to 18–24 months. Only one patient (11%) relapsed after discontinuation of MMF and was successfully retreated. Ureteral stenting was performed in seven patients (78%). None of the patients underwent ureterolysis or nephrostomy tube insertion. Clinical improvement occurred in eight patients (89%), while one (11%) had a variable response.

Renal function stabilized or improved in all patients (100%). Complications occurred in five patients (56%): AKI in two (22%), persistent unilateral renal nonfunction in two (22%), and CKD in one (11%).

### Radiologic outcomes and quantitative measures

All nine patients (100%) presented with hydronephrosis, bilateral in eight (89%) and unilateral in one (11%). Hydronephrosis grade was documented in 8/9 patients; grade improved by at least one grade in 6/8 patients and resolved completely in 3/8 patients.

### Temporal trends in management

The center’s core strategy remained corticosteroid-based induction with consideration of a steroid-sparing agent and early ureteral decompression when indicated across the study period. A shift toward preferential use of MMF was observed from 2018 onward; no patients in this series received biologic therapy during the study interval.

## Discussion

Idiopathic retroperitoneal fibrosis is a rare fibro-inflammatory disease characterized by the development of dense retroperitoneal tissue, often leading to ureteral entrapment and renal impairment^[^1,5^]^. Our case series of nine patients illustrated the clinical variability of this condition and contributed observational data on outcomes and treatment strategies, particularly the combined use of corticosteroids and immunosuppressive drugs.

The most common presenting symptom in our cohort was flank or abdominal pain, which aligned with prior studies identifying this as the hallmark manifestation. Several patients also experienced constitutional symptoms such as fatigue and weight loss. Biopsy was performed when malignancy was suspected.

There is no standardized treatment at present, and several protocols have been used with no clear advantage of one over the other^[^8^]^. Besides corticosteroids, several drugs have been used including MMF, azathioprine, methotrexate, cyclophosphamide, rituximab, cyclosporine, tocilizumab, immunomodulators (colchicine), and anti-hormonal agents (tamoxifen)^[^8,13^]^. Mycophenolate mofetil showed good effect in our experience with minimal side effects.

Notably, one patient who declined immunosuppressive therapy experienced spontaneous improvement in both symptoms and imaging, demonstrating that a self-limited disease course can occur in select cases of IRF. This observation supports a cautious, individualized approach for patients with mild symptoms, preserved renal function, and no radiologic signs of rapid progression. It also justifies considering an initial conservative strategy consisting of prompt ureteral decompression when indicated, close clinical and biochemical surveillance, and short-interval imaging before committing to long-term systemic immunosuppression in carefully selected patients. Clinicians should document objective baseline measures (renal function, inflammatory markers, and standardized imaging dimensions), schedule frequent reassessments during the watchful period, and move promptly to immunosuppressive therapy if symptoms, renal function, inflammatory markers, or imaging worsen.

Although renal function improved in most cases in our series, persistent impairment in two patients underscored the risk of chronic kidney injury with delayed diagnosis or irreversible obstruction. In case of treatment failure and/or severe diseases, surgical interventions may be needed. An occasionally employed treatment approach is ureterolysis, which involves the release of the ureters and can be performed either laparoscopically or through open surgery^[^14^]^. Seven of our patients required urologic interventions with ureteral stenting, and none underwent ureterolysis or insertion of nephrostomy tubes.

Radiological imaging was essential not only for diagnosis but also for monitoring fibrosis regression and hydronephrosis. In addition to sonography, CT, MRI, and PET scan may also be utilized as a diagnostic modality and for follow-up^[^15^]^.

Inflammatory markers (ESR, CRP) generally paralleled clinical response, although their specificity remained limited. Autoimmune markers were variably positive, which highlighted the clinical overlap and diagnostic challenges between IRF and other immune-mediated disorders such as IgG4-related disease^[^2,16^]^.

Two patients in our cohort were pediatric. One had multiple comorbidities that raised concern for a secondary, rather than idiopathic, retroperitoneal fibrosis. The second patient was otherwise a healthy female with no significant comorbidities.

Idiopathic retroperitoneal fibrosis is extremely rare in the pediatric age group and warrants heightened diagnostic vigilance because secondary causes and mimics are more common in this population. Reported pediatric cases demonstrated that children can present with the same clinical spectrum as adults with flank or abdominal pain, constitutional symptoms, and obstructive uropathy leading to hydronephrosis^[^17,18^]^. Secondary etiologies (post-infectious, post-radiation, drug-related, vasculitis, or syndromic fibroinflammatory disorders) must be actively excluded with targeted imaging, histopathology including immunohistochemistry for anaplastic lymphoma kinase and IgG4 when indicated, microbiologic studies, and genetic testing when clinically suggested^[^19^]^. MRI with delayed post-contrast sequences is particularly useful in delineating the extent of fibrosis and monitoring response without ionizing radiation^[^17^]^. Management parallels adult practice with ureteral decompression for obstruction and corticosteroid-based medical therapy supplemented by steroid-sparing agents when indicated, yet the decision to biopsy and to initiate systemic immunosuppression in children should be individualized within a multidisciplinary team given the rarity of idiopathic cases and the higher pretest probability of alternative diagnoses.

Emerging biologic therapies have shown promise for steroid-refractory or relapsing retroperitoneal fibrosis. B-cell depletion with rituximab has been associated with significant radiologic shrinkage and symptomatic benefit in several series and case reports, including a cohort demonstrating a statistically significant reduction in lesion diameter after rituximab treatment^[^9,10^]^. Interleukin-6 pathway blockade with tocilizumab has also been reported to induce clinical and radiologic improvement in refractory chronic periaortitis/retroperitoneal fibrosis in a case report, supporting IL-6 as a potential therapeutic target in selected patients^[^20^]^. Although these findings are encouraging, the evidence is derived predominantly from small retrospective series, case reports, and single-center experiences; randomized controlled trials comparing biologic agents with standard corticosteroid ± steroid-sparing regimens are therefore required to establish comparative efficacy, safety, and optimal patient selection.

This study is limited by its retrospective design, small sample size, and heterogeneous follow-up. Nevertheless, in this nine-patient series of IRF, early multimodal imaging combined with corticosteroid plus immunosuppressive therapy (prednisone with MMF or azathioprine) led to clinical improvement in 89%, radiologic regression in 67%, and stabilization of renal function in all patients. However, the high rate of complications and long-term sequelae underscores the need for individualized management and close monitoring.

These findings underscore the importance of prompt ureteral decompression and individualized immunosuppressive regimens in preserving renal function and reducing morbidity in routine clinical practice. Looking ahead, prospective multicenter trials are needed to refine treatment duration, evaluate emerging biologic agents, and establish standardized, evidence-based management algorithms for IRF.

## Data Availability

Deidentified individual participant data that underlie the results reported in this article (including demographics, clinical presentation, laboratory values, imaging findings, treatments, and outcomes) and the study protocol will be made available upon reasonable request to the corresponding author. Data sharing will commence after publication and remain available for 3 years. Requests must include a methodologically sound proposal and will require a data-use agreement in accordance with institutional ethics approval.

## Research registration unique identifying number (UIN)

Not applicable. This is a case series of nine patients.
